# Nine quick tips for analyzing network data

**DOI:** 10.1371/journal.pcbi.1007434

**Published:** 2019-12-19

**Authors:** Vincent Miele, Catherine Matias, Stéphane Robin, Stéphane Dray

**Affiliations:** 1 Université de Lyon, F-69000 Lyon, Université Lyon 1, CNRS, UMR5558, Laboratoire de Biométrie et Biologie Évolutive, Villeurbanne, France; 2 Laboratoire de Probabilités, Statistique et Modélisation, Centre National de la Recherche Scientifique, Sorbonne Université et Université de Paris, Paris, France; 3 UMR MIA-Paris, AgroParisTech, INRA, Université Paris-Saclay, Paris, France; University of Toronto, CANADA

## Introduction

From the molecular to the ecosystem level, a biological system can often be represented as a set of entities that interact with each other. Recent advances in data acquisition technology (e.g., high-throughput sequencing or tracking devices) open up the opportunity to quantify these interactions and call for the development of ambitious methodology to tackle these data. In this context, networks are widely used in biology, bioinformatics, ecology, neuroscience, or epidemiology to represent interaction data [1]. A network contains a set of entities (the nodes or vertices) that are connected by edges (or links) depicting some interactions or relationships. These relationships may be either directly observed or deduced from raw data. The first case encompasses protein–protein interaction (PPI) networks—in which interactions between 2 proteins are experimentally assessed—or plant–pollinator interactions that are directly observed in the field. Gene regulatory networks reconstructed from gene expression data, co-occurrence networks inferred from species abundances, or animal social contact networks deduced from Global Positioning System (GPS) tracks are some examples of the second case. New kinds of networks are still emerging (for instance, cell–cell similarity networks [2], Hi-C networks, and image similarity networks [3]).

Networks are very attractive objects, and many methods have been developed to analyze their structure. However, biological networks are often analyzed by nonspecialists, and it may be difficult for them to navigate through the plethora of concepts and available methods. In this paper, we propose 9 tips to avoid common pitfalls and enhance the analysis of network data by biologists.

## Tip 1: Formulate questions first; use networks later

Network theory is well established and truly powerful, but it cannot be used as a "black-box." Indeed, building a network should not be considered as an end in itself. We recommend (1) establishing a list of scientific questions and hypotheses before manipulating the data, and then (2) evaluating whether these questions naturally translate into a series of network analyses, rather than making network analyses first and checking whether they raise questions after (in agreement with Rule 1 in [4]). Indeed, it is generally immediate to represent and model the data with a network but much trickier to translate a question into a network-based analysis.

To this end, besides integrating the network formalism, it is important to embrace the network viewpoint. It relies on a cornerstone idea that makes the strength but also the challenge of network modeling: Any interaction is considered within its context, taking into account the other interactions that occur (or not). In this viewpoint, any interaction between 2 nodes is considered not only in the context of other pairs involving these nodes but also in relation to the global connectivity pattern. For instance, the importance of a particular edge between 2 genes will be differently assessed if the target gene is or is not a hub (i.e., regulated by many genes). This viewpoint does not consider interactions as independent objects and is thus the exact opposite of examining the set of interactions one by one.

Finally, it is obviously recommended to check whether your questions and data really fit the network viewpoint before performing any analysis. If the number of nodes and/or edges is very low, network analysis can be applied, but results can be disappointing because there are not enough observed interactions to identify a structure in the data. On the other hand, although any matrix can be viewed as a network (1 edge per cell; see next tip), it is often more adequate to consider using nonnetwork methods dedicated to complete matrices. For instance, a correlation matrix, possibly viewed as a correlation network, can be naturally analyzed with a hierarchical clustering or a principal component analysis. In other words, network analysis is not necessarily the answer when analyzing a data matrix.

## Tip 2: Categorize your network data correctly

To grab the cutting-edge concepts and methods in the networks field, learning the appropriate vocabulary from graph theory is a prerequisite [5]. In particular, it is important to categorize your network properly to be sure you apply suitable methods. Different network categories for different data lead to different approaches. Edges can be directed (from a source to a target), possibly including self-loops (e.g., a protein interacting with itself or cannibalism in food webs). Ignoring this information for the sake of simplicity would actually betray the original data. When dealing with edges embedding a value (a weight), we advise you to avoid transforming the network into a binary one, disregarding the weights or keeping only the edges with weight above or below a certain threshold. Indeed, it clears a significant part of the available information because some aspects of the network structure might be undetected in the binarized network [6]. It would therefore be naive to consider that analyzing a binarized network or the original weighted one is roughly equivalent. Moreover, methods handling weighted networks are generally available and therefore more appropriate. However, in some instances, it is actually useful to study the weighted and binary versions separately, to be able to disentangle 2 effects driving network structure: interaction occurrence (presence or absence) and intensity (weights). For example, some authors have reported that a nested pattern was frequently observed in binarized ecological networks but not in weighted ones [7]. Lastly, the data analyst must be very cautious since, in the literature and in the available methods, weights can be considered as intensity based (the greater the weight, the stronger the edge is) as well as distance based (the smaller the weight, the closer the nodes are).

Nodes can belong to different categories, and edges can be allowed only between nodes of different categories (bipartite, tripartite, and multipartite networks; e.g., nodes as hosts and parasites, or as plant, fungus, and seed dispersers [8]). It is mandatory to select methods that handle this particularity. For instance, many statistical approaches rely on the expected number of edges (e.g., in the computation of modularity, see Tip 5), which is here clearly different compared to the unipartite case.

Finally, additional information on the nodes is often available. For instance, nodes can have spatial positions (e.g., nodes as habitat patches or farms in 2D and brain area in 3D) or can be associated to external attributes (e.g., species traits in a food web). This additional information can be explicitly considered in the analysis, either to understand whether it contributes to organize the network [9] or to look for some remaining structure once accounted for its effect (e.g., spatial [10] or phylogenetic effect [11]). In the former case, a simpler but suboptimal alternative often consists in using this information a posteriori in the interpretation of results (e.g., explaining the structure of genetic networks with spatial information [12] or comparing network structure with metadata [13]).

## Tip 3: Use specific network analysis software

A range of versatile software is dedicated to network analysis. It is therefore a waste of time trying to use unspecific tools. These software tools belong to 2 distinct categories that have pros and cons: graphical user interface (mouse-based navigation) and software packages (command line interface or programming). The first category is mainly dedicated to powerful and interactive visualization (see Tip 4). It includes the 2 major open source software tools, Gephi and Cytoscape, both supported by an active community. They also offer the computation of some network metrics (the choice of a relevant metric is discussed in Tip 5). The second category is dominated by the 2 leading general-purpose network packages, NetworkX and igraph, but there exist plenty more specific packages (for instance, bipartite in R). Browser-based visualization [14] recently emerged as an intermediate category, mostly based on a collection of JavaScript libraries (e.g., Sigma.js).

That said, we strongly suggest that you learn programming and scripting your analysis (in agreement with papers in the "Ten Simple Rules" collection about computing skills and reproducibility [15, 16]). Dealing with reproducible code enhances network research: You can rerun with no effort the complete analysis on a modified version of your raw data on different datasets and share the code with other colleagues interested in the modeling approach. Finally, there exists a limited set of common network file formats (e.g., adjacency list in the format source target) that you should adopt from the very beginning to easily switch between different software tools.

Meanwhile, the data analyst should avoid a hasty use of the different functions implemented in these tools. As underlined in Tips 5 and 6 it is crucial to understand the metrics and methods before running functions and to select the appropriate ones with respect to the questions and the data at hand.

## Tip 4: Be aware that network visualization can be useful but possibly misleading

One powerful aspect of networks is their ability to depict complex data in a single object. It can therefore be natural to represent networks graphically in 2 dimensions: Nodes are spread in the plane and edges drawn accordingly, with the objective to achieve the most aesthetic and informative design [17]. Before we go any further, as nodes' positions (called a graph layout) in such a display are not part of the data but result from a particular choice or method, we encourage biologists to clearly describe the layout used in any graphical representation of a network in scientific publications, especially to make it reproducible.

**Fig 1:**
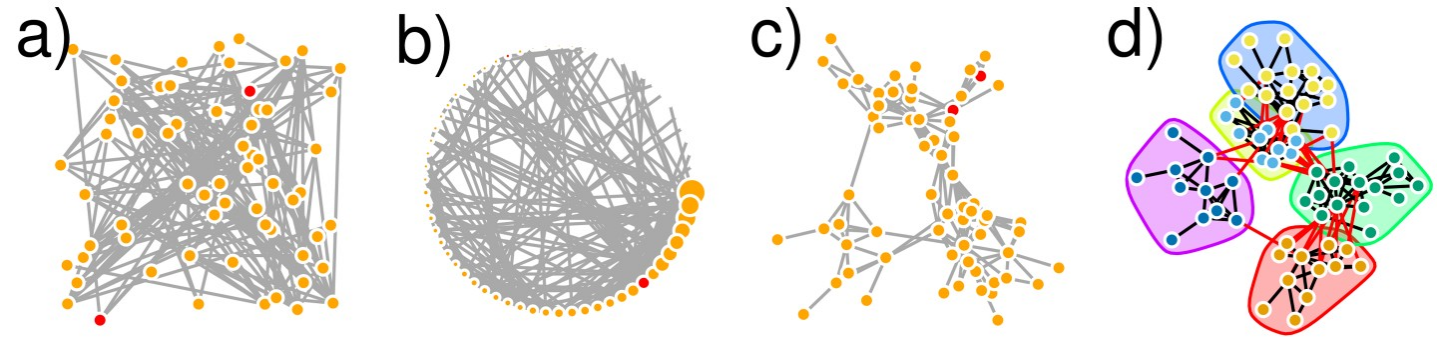
Four visualizations of the same network modeling interactions between 64 sociable weavers [14, 22]. (a–c) The same 2 nodes are colored in red to show that their distance varies depending on the layout. (a) Random layout. (b) Circle layout in which nodes' size and position are defined by their degree. (c) Fruchterman and Reingold layout, showing 3 apparent clumps on top and bottom right and left. (d) Kamada and Kawai layout set with weights on edges (in red) connecting the 5 clusters obtained with the Louvain algorithm (see Tip 6 and [23] for details). The clusters are delineated by different colours. Performed with the R package igraph.

**Fig 2:**
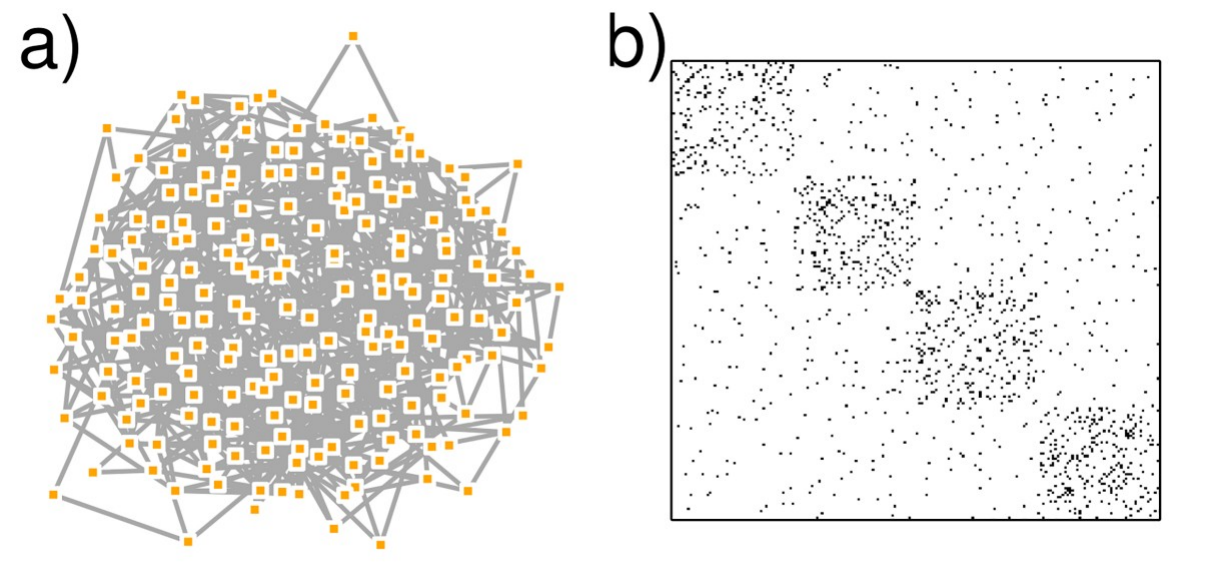
Synthetic network with 200 nodes and 700 edges generated with an SBM (see Tip 6) with 4 clusters of intraconnectivity and interconnectivity of 0.1 and 0.015, respectively. (a) Network visualization with Kamada and Kawai layout does not highlight any modular structure, whereas it exists. (b) Representation of the adjacency matrix with row and columns ordering consistent with the 4 clusters. Performed with the R package igraph. SBM, stochastic block model.

Graphics are usually considered as an important tool for exploratory data analysis [18]. An active research community proposed a series of heuristics (available in the tools mentioned in Tip 3) aimed at obtaining a nice network view in a reasonable time, despite the growing size of available networks. This apparently simple task is in fact a very hard combinatorial problem and consists of searching for the optimal layout for a given set of objectives that you often ignore (e.g., maximizing attractions between connected nodes or minimizing edge crossings). As a consequence, what you see with your eyes can be biased. Indeed, special care is required to not overinterpret network visualization when exploring the data. For instance, always keep in mind that the distance between 2 nodes should not be interpreted as an intrinsic measure of proximity because another display algorithm would result in a possibly very different distance (see 2 red nodes in Fig 1A–1C). Moreover, it is better to avoid hasty conclusions drawn on the sole basis of a network visualization (e.g., Fig 1C could suggest a modular structure with 3 clusters, whereas a rigorous network analysis could conclude 5 clusters as represented in Fig 1D). On the other hand, if no structure arises from a visual inspection of the network in an explanatory step, it does not mean that further network analysis is not necessary (see Fig 2A), especially when dealing with large networks.

On the other hand, network visualization can be useful as a way to illustrate the results of a network analysis (as presented in Tips 5 and 6). In this case, a layout should be chosen for its ability to highlight network properties (degree heterogeneity in Fig 1B) or conclusions drawn by an analysis (Fig 1D). For instance, nodes can be positioned according to the values of some particular metrics of interest [19]. However, a network illustration must not only be aesthetic, it has to be informative about the nodes as well as the edges structure. We therefore encourage the analyst to carefully consider the messages to be conveyed through a network illustration. For instance, it is frequent that bipartite ecological networks are illustrated with 2 stacked layers (e.g., in bipartite package); in some cases, these illustrations only highlight nodes' information (how many edges they are involved in) but usually fail to show any edge's structure due to the many crossing edges inherent to this representation.

Lastly, we also advise considering visualizing the adjacency matrix as a heatmap or a colored matrix (see Fig 2 in [20] for an explanation). It allows one to represent the presence or weight of edges (colored cells), but it also has the advantage of highlighting edges' absence (blank matrix cells). This is particularly relevant when the matrix rows and columns are reordered in an informative manner (e.g., by increasing value of a metric [21] or according to some clustering results; see Tips 5 and 6 and Fig 2B).

## Tip 5: Avoid blind use of metrics; understand formulas instead

Beside the limitations of network visualization, describing a network can also (and advantageously) consist of computing summary statistics. The beginner will immediately find the path to a series of network metrics: one number per node or edge (local metrics; e.g., degree) or one number for the whole network (global metrics; e.g., connectance/density or modularity). Metrics have proliferated, and it is strongly advised to take time to read carefully the mathematical definition of the metrics one has at hand (see also Tip 9): The deeper the mathematical understanding, the easier the interpretation is. For instance, the concept of nodes' centrality goes with a range of centrality metrics that have different meanings. Moreover, it is so easy to compute any metric with the aforementioned software tools that it can sometimes prevent the analyst from checking their pros and cons. As an example, reading the definition of the widely used betweenness centrality, you can understand that it is based on shortest paths. If you intend to use this measure, it is therefore necessary to check whether the shortest path is a relevant concept associated to the process under study (such as energy fluxes in food webs) or whether it is more questionable (e.g., paths in functional networks may not actually correspond to information flow [20]; paths in contact networks may not be relevant when information or disease diffusion is not studied [24]). Another example consists in the analysis of directed and/or weighted networks with extensions of metrics to this case. It is important to note that the formula of the weighted degree accounts for 2 effects: how many neighbors and how large the weights are, 2 effects that are impossible to disentangle (a weighted degree of 2 can correspond to a single edge of weight 2 or 4 edges of weight 0.5 each). A similar problem can also be raised for the weighted path (potential pitfalls highlighted in [25]). Lastly, global metrics are often used to compare networks (networks measured from different data or conditions or simulated networks as mentioned in Tip 7). In this case, special care should be taken when comparing values because metrics differences can be a side effect of differences in simple network characteristics such as the number of nodes or edges (see common pitfalls mentioned in [26] for brain networks and a discussion on covariation of metrics with characteristics of ecological networks in [27]). For instance, modularity, number of modules, and network size are known to be intertwined [28].

It is not unusual that authors, instead of choosing a given metric adapted to a particular question, compute a high number of metrics among the available ones. However, many metrics are correlated (see a correlation study in [24]), and it becomes necessary to deal with this redundancy to interpret the results (e.g., with an ordination method [29]). This approach is not hypothesis driven as recommended in Tip 1 and can undeniably be replaced by an incremental approach in which metrics are selected one at a time for their ability to check particular hypotheses associated to the fundamental questions on the data (as for many statistical analyses, see rule 5 in [4]).

## Tip 6: Avoid blind use of clustering methods; check their difference instead

With the data avalanche arising this decade, leading to larger networks, clustering has become one of the most popular tools to get a comprehensive view of the network structure. Its general purpose is to aggregate nodes into clusters in order to identify a meso-scale structure in the network (i.e., zooming out the network). Choosing a network clustering raises issues similar to choosing a network metric (Tip 5). It is much more than using one of the functions available in a software. As for clustering methods on point clouds, the ones constructed on networks aim at gathering similar objects (i.e., nodes) and thus rely on a specific definition of node similarity. What does the analyst want to be similar in a network? Discussing the pros and cons of the different methods is beyond the scope of this article, whereas a massive literature on the topic exists (see Tip 9). However, we illustrate the impact of choosing a specific definition for node similarity with 3 classical proposals (among others).

A first and natural definition for the similarity between nodes is the existence of a connection between them. Based on this definition, network clustering consists in finding a modular structure, i.e., identifying dense clusters of nodes (also called modules or communities) poorly connected with others. Community detection methods [23] implement this approach, which implicitly assumes the existence of modules in the network. They were successfully applied in many studies in biology (for instance, to identify chromatin domains [30]). A second approach considers that 2 nodes are similar when they tend to be connected (or unconnected) with the same type of nodes. Hence, species in a food web are considered similar if they have similar preys and predators [31]. This definition can accommodate networks with nonmodular structure [32] since it assumes that the nodes are involved in a "diversity of meso-scale architectures" [33]. The stochastic block model (SBM) is a popular method based on this definition [32, 34], which has shown to be relevant for the analysis of some biological networks (to highlight the complex architecture of connectomes [33] or functional groups in ecological networks [35]). One important feature is that it allows one to model explicitly edge directions and weights by means of different statistical distributions [11]. A third approach consists in associating a vector of characteristics to each node and then gathering nodes with similar characteristics. This includes motifs-based approaches [36] and a wide range of innovative node-embedding techniques [37, 38]. Nodes are described as points in a space with reasonable low dimension, which allows one to apply the huge variety of existing clustering methods for multivariate data. It is important to realize that each of these similarity concepts naturally results in different nodes clustering. The choice between these alternatives must be driven by biological questions, not by their availability in software tools (Tip 1).

## Tip 7: Don't choose the easy way when simulating networks

To highlight the properties specific to an observed network (for instance, a peculiar metric value), a common practice consists of comparing with simulated networks. These properties are detected as a significant deviation (or not) from a typical behavior implemented in simulated networks. However, there is no generic definition of a typical network and, as a consequence, the features that can be detected depend dramatically on the null model used to simulate networks. This null model must be chosen for a given purpose, fitting expected behaviors, while contrasting those we are interested in. In other words, it must fit the data reasonably well to avoid numerous false discoveries, but not too well so that deviations can emerge.

A natural option could consist of selecting a null model among the series of random graph models (e.g., Erdős–Rényi, small-world, scale-free, SBM, exponential random graph, or configuration model). However, we recommend not to use them too hastily because they are often too general. For example, the Erdős–Rényi model (all edges independent and having the same probability of occurrence) is often a poor null model to detect nodes having an unexpectedly high degree. Indeed, it induces a Poisson degree distribution, which is so far from the one observed in most networks that many nodes appear to be unexpectedly connected. On the other hand, no node can display an unexpectedly high degree with respect to the configuration model, as this null model precisely fits to the degree of each node. Moreover, the analyst is usually aware of a series of properties that should be displayed by a simulated network: imbalanced degree distribution, different nodes' roles associated with available side information, forbidden interactions (e.g., depending on body mass in food webs [39]), etc. Such expected properties must be encoded in the simulation process (for instance, a fixed degree sequence [35]), otherwise they will emerge and be detected as significant or contribute to detecting false significant effects as side effects. As an example, when assessing whether the number of feed forward loops is unexpected in a given transcription network, the simulation procedure must rely on a fixed number of nodes and degrees, whereas the number of these loops remains free.

Lastly, when the network under study is not directly observed but built from raw data interpretation, it can be relevant to simulate the whole construction process. Consider the case of contact networks inferred from movement data [24]: One can either simulate trajectories keeping some properties of the original data and then build a contact network or directly simulate a "realistic" contact network. The former approach will intrinsically account for the uncertainties and biases induced by the construction steps, which are likely to be overlooked by the latter approach.

## Tip 8: Reconsider the data to build multiple network layers

A network object can be the result of data aggregation. Indeed, interactions are often observed at different times and locations or in different conditions. You are therefore strongly urged to keep in mind (and at hand) the different layers of data (time, space, type, etc.) and consider networks composed of multiple layers, because multilayer networks can provide new insights compared to an aggregated one [40–42].

A network is called dynamic when it gathers a time series of network snapshots corresponding to successive rounds of data collection (the nodes' list possibly varying in time). In this case, the temporal variability of the network structure can be assessed (e.g., rewiring of interactions or changes in network metrics over time), and extensions of the concepts developed in Tip 6 now exist in the dynamic case [43, 44]. For instance, the dynamics of animal social structure can be inferred from dynamic networks to enhance the understanding of disease transmission [45]. On the other hand, interactions can be observed at different spatial locations. In ecology, they are often aggregated in a metanetwork (or metaweb [46]) to study how the local networks differ from this metanetwork and explain these variations with environmental factors. In these 2 cases, multiple layers allows one to describe a network as an evolving object, and the analysis aims to identify the spatiotemporal variations of interactions and their drivers.

Different kinds of interactions can also be observed between nodes. Stacking layers representing molecular interactions in different human tissues [47] or mapping extrasynaptic and synaptic connectomes [48] leads to a multiplex network: Between any 2 nodes, there possibly exist more than 1 edge, 1 per interaction type at most (often visualized with different colors). Taking jointly into account the different layers enhances the understanding of the nodes' interplay. For instance, using jointly trophic and nontrophic interactions enhances the definition of species’ ecological roles compared to the use of single layers independently [35]. Finally, it is also possible to integrate different layers of information with different sets of nodes for each layer, such as proteins and chemical compounds [49]. In this case, different kinds of interactions are defined inside and between layers. In all these cases, different information layers are integrated into a comprehensive network such that they are treated jointly rather than one after the other.

## Tip 9: Dive into the network literature beyond your discipline

Network science now involves a hyperactive community of researchers from different domains such as physics, statistics, computer science, or social science. As a result, a massive literature on networks exists, and it is challenging for biologists to dive into it. Indeed, we are not used to exploring the bibliography outside our research domain. Reference books [5, 42, 50, 51] and reviews [23, 40, 52] are obviously good entry points for developing your network skills. However, without any doubt, you will highly benefit from a round trip in this literature exogenous to your field (including the most recent advances in network methodology available in the arXiv preprint repository), provided that you make the effort to learn the appropriate vocabulary of this area. Concrete examples include the analysis of modularity in biology, which was borrowed from physics (unlike nestedness, which originates in biogeography), or the recent use of SBMs (Tip 6) that have been applied in the social science literature since the last century.

## Conclusion

The 9 tips presented here should be a way for the data analyst to get a foot in the door of network data analysis. These tips are not exclusive, and we are aware of other network-based questions that deserve special interest, including diffusion on networks, for instance. Still, the network nonspecialist must be confident in his or her ability to learn, step by step, the network concepts and methods with a productive effect on his or her scientific questions.
